# Supervised satellite classification and field validation document endangered *Acropora* collapse, and identify surviving colonies in Belize

**DOI:** 10.1038/s41598-026-47081-w

**Published:** 2026-06-02

**Authors:** Lisa Greer, Raja Das, Harris Foad, Karl Wirth

**Affiliations:** 1 Department of Earth and Environmental Geoscience, Washington and Lee University, Lexington, VA USA; 2 Department of Geology, Macalester College, St. Paul, MN USA

**Keywords:** Coral, Belize, *Acropora*, Remote sensing, Refugium, Conservation, Climate sciences, Ecology, Ecology, Ocean sciences

## Abstract

*Acropora cervicornis* and *Acropora palmata* have long provided structural complexity to Caribbean reefs, yet they have declined by over 90% in recent decades. Identifying places where wild populations of at-risk corals can survive remains essential for conservation. Coral Gardens, Belize, has historically been a refugium for endangered *Acropora spp*., but mass mortality transpired in 2023. This study aimed to determine whether coral loss at a small long-term field monitoring site was representative of regional trends. We developed a supervised Random Tree classification model to identify the spectral signature of *Acropora spp*. in satellite images and predict live coral cover across 25 km² from 2011 to 2023 to compare with field data from 2012 to 2025. Model data predicted a drop in live *Acropora spp*. of over 90% from 2011 to 2023 coincident with total loss at the field site. Field validation of the model became a search for survivors in June 2025. Live or recently dead *Acropora spp*. were found at 28 of 42 predicted sites, and live colonies were found at 16 previously undocumented locations. This methodology provides a tool for monitoring shallow-water *Acropora spp*. and locating survivors. Our findings underscore the vulnerability of even persistent coral refugia.

## Introduction

*Acropora cervicornis* and *Acropora palmata* coral have been critical reef building species in the Western North Atlantic and Caribbean since the Pleistocene^1–3^. These fast growing Scleractinian corals have historically provided the architecturally complex scaffolding needed to sustain ecologically diverse habitats in shallow marine coral reef environments^4–7^. *A. cervicornis* grows up to 5 to 10 times the rate of other Caribbean coral species, with linear extension rates ranging from ~ 10 cm to over 35 cm a year^8, 9^. Individual branches on the order of up to a few cm in diameter can form dense thickets in shallow water (< 8 m depth). The high growth rate and branching nature of this species facilitates rapid expansion of coral reef space. The thicker, larger, and sturdier *A. palmata* can grow up to just below sea level, with linear extension rates up to 9 cm a year^10^. It is often found close to the reef crest along the Mesoamerican barrier reef, playing a critical role in wave attenuation for coastline communities. Both sibling acroporid species as well as *Acropora prolifera*, a smaller and more delicate branching hybrid of *A. cervicornis* and *A. palmata* that likely evolved in the Pleistocene^11^, are commonly found in shallow water lagoonal patch reefs inshore of the reef crest. Small colonies of *A. cervicornis* can also be found in deeper water on the forereef^12^.

Not only have *Acropora spp*. long dominated many Caribbean reefs, they have persisted through adverse climate and environmental changes in the geologic past^13–^^16^. But since the 1970’s and as far back as the 1960’s, *Acropora spp.* have experienced population declines of up to 98% across the Caribbean^8,15^ with particularly acute losses documented in 2023 in certain areas, for example Florida^17^. *A. cervicornis* and *A. palmata* were listed as critically endangered by the International Union for Conservation of Nature and Natural Resources in 2008^18^. The reasons cited for this dramatic decline vary but are often synergistic and frequently attributed directly to anthropogenic causes^19^. White Band Disease^1,20^ clearly caused extensive damage to *Acropora spp.* corals across the Caribbean. The prevalence of this disease might also have been exacerbated by additional stressors to the corals^21,22^. Macroalgal competition due to nutrient pollution or overfishing^23^ and recreational damages^24,25^ have been shown to negatively impact *Acropora spp*. Ocean acidification may already impact *Acropora spp*. population dynamics or growth at some locations, with increasing impacts projected^26–28^. Despite multiple threats to coral health, coral bleaching, or the expulsion of symbiotic zooxanthellae from coral tissue as a direct result of temperature stress due to anthropogenic climate change, is the leading cause of coral mortality across the globe^17,29^.

As global temperatures continue to rise and additional threats to reefs continue, finding, conserving, and protecting remaining wild reefs becomes imperative. Great strides have been made in breeding and cultivating *Acropora spp*. in a laboratory setting^30,31^ and multiple efforts to fragment and outplant *Acropora* species in the field have found success^32–34^. But restoration is resource-intensive in labor and cost, therefore limiting the reach of individual projects^35–37^. Targeted engineered efforts to replace coral following an acute loss at a once healthy site, like a boat grounding or storm, can be expensive and require extensive monitoring^38^. Coral gardening (farming and transplanting corals from nursery sites) has resulted in great success locally but choosing sites that are naturally favorable for growth are critical to success^34,35,39,40^. Evidence-based *Acropora spp*. restoration strategies should center on sites that show potential for coral resilience and methods appropriately adapted to those sites to best achieve ecological function and increased coral biomass^41,42^. Finding sites where *Acropora spp*. coral have been documented to thrive is increasingly challenging. Coral Gardens, Belize has served as an area where *A. cervicornis* populations continued to thrive during a period of Caribbean-wide population decline.

**Fig. 1:**
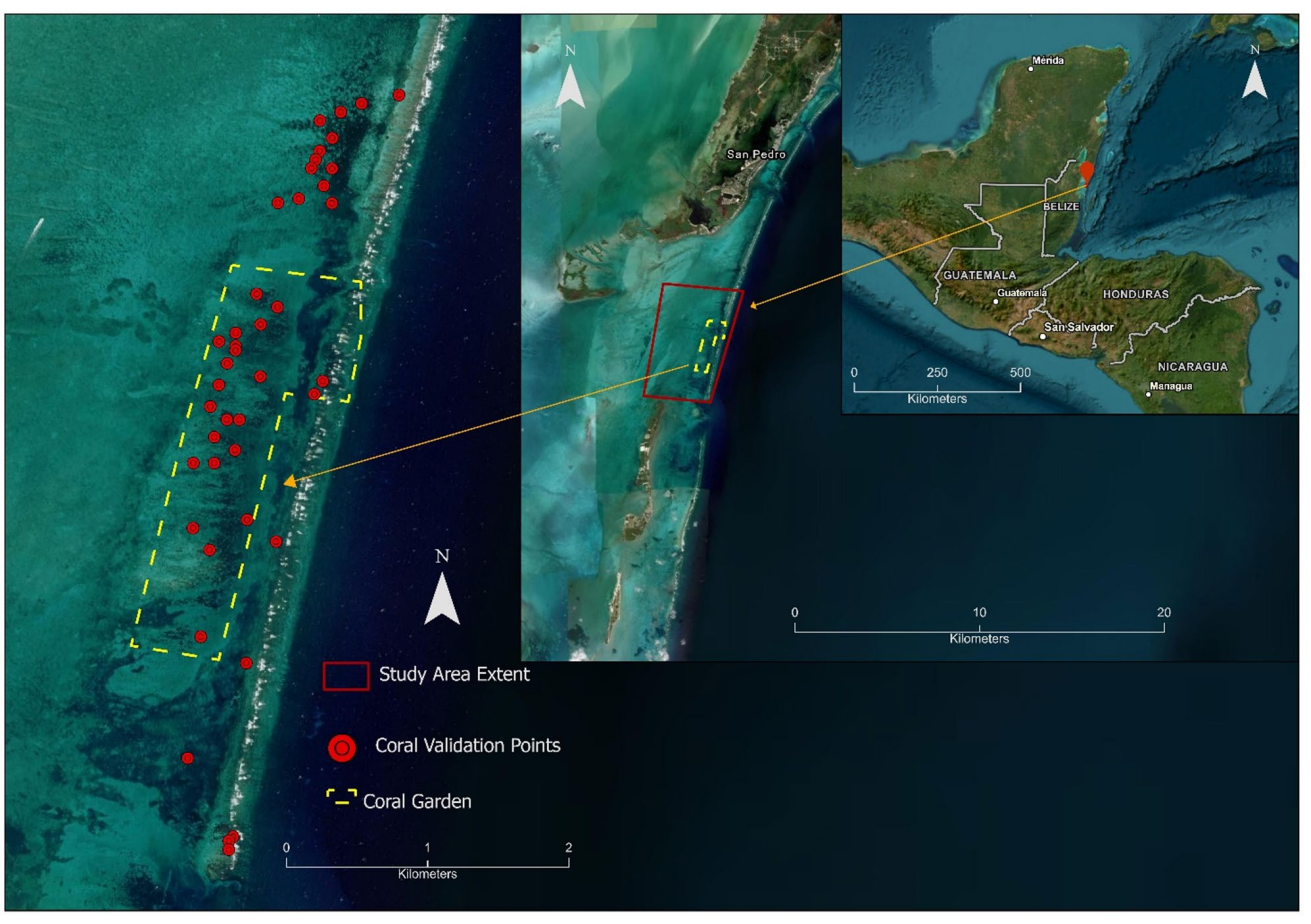
Map showing the study area off the coast of Belize. The red and yellow boxes (in the center image) show the study area extent and Coral Garden locations, respectively. The dark red points indicate locations visited during field survey. Insets provide regional context within Central America (top right) and the location of the site within the Belize Reef system (center and left). Map generated using ArcGIS Pro version 3.3.0 (https://www.esri.com/en-us/arcgis/products/arcgis-pro/overview).

Coral Gardens is loosely defined by a large system of shallow water (< 7 m depth) lagoonal patch reefs between Ambergris Caye and Caye Caulker behind the Mesoamerican reef crest (Fig. 1). Previous work on Coral Gardens began with field surveys in 2011 and continued at least once annually through June 2025. Each assessment included detailed photo documentation of the same small ~ 200 m^2^ area of Coral Gardens. Each year ~ 146 m^2^ quadrats were placed along 5 established transects and photographed, images were scaled, and all live *A. cervicornis* was manually traced to track percent live coral following methods outlined in Greer et al., (2023). In 2016, an unsupervised classification model was used to map *Acropora spp*. populations within Coral Gardens^43^. To understand whether the exceptional *A. cervicornis* growth at Coral Gardens was ephemeral or long-lived, Irwin et al.^44^ employed an unconventional dating method which used the somatic mutation rates in microsatellite markers to estimate the age of each coral genet found at Coral Gardens using methods developed by Devlin-Durante et al.^45^. The genetic data suggested that veteran colonies as old as 409 to 561 years of age lived alongside new sexual recruits at this site. An additional study designed to test the age of *A. cervicornis* using traditional radiocarbon and uranium-series techniques supported the conclusion that coral growth had been persistent and uninterrupted at Coral Gardens during wider Caribbean-wide decline^46^. Together these studies documented that Coral Gardens was one of the few known refugia for *A. cervicornis* left in the Caribbean^46^. Subsequent work suggested that a lack of anthropogenic stressors in this area facilitated the ongoing success of *Acropora spp*. coral at Coral Gardens and that increasing sea surface temperatures (SST) posed the most urgent threat to Coral Gardens^47^. Monitoring the health of this rare refugium may be important for understanding changes in endangered *Acropora spp*. populations. But field-based study can be costly, time consuming, labor intensive and limited in reach.

Over the past three decades, remote sensing has emerged as a vital tool in coral reef science, revolutionizing our ability to monitor reef systems at large spatial scales. It enables cost-effective, repeatable observations over remote and often inaccessible areas, addressing many of the limitations of traditional field-based surveys^48–52^. Early studies using moderate- to low-resolution satellite imagery, such as Landsat, demonstrated the feasibility of distinguishing coral reefs from surrounding substrates like algae, sand, and seagrass based on their distinct spectral signatures^50,53–56^. However, low- to moderate-resolution satellite imagery often fails to capture the fine-scale heterogeneity of benthic environments due to its coarse spatial resolution, leading to spectral mixing that blurs or averages the distinct spectral signatures of individual habitat components^57^. This sub-pixel mixing is one of the primary reasons for reduced mapping accuracy, as it obscures the reflectance differences needed to discriminate between features such as live coral, bleached coral, dead coral, and algae. Additionally, water column effects such as water clarity, turbidity, and the depth of the water column can further distort the spectral signatures of benthic features, reducing their distinguishability in low- to moderate-resolution satellite imagery^57^.

In contrast, high resolution satellite imagery enables detection of detailed benthic habitat. Since high resolution pixels cover smaller areas, they contain purer signals of individual benthic types, reducing the spectral mixing that confounds classification in coarser pixels. Wilson^58^ compared WorldView-3 (2 m, 8 bands) and Sentinel-2 (10 m, 4 bands) for coastal bottom habitat mapping in Atlantic Canada and found that WorldView-3 yielded significantly higher classification accuracy (> 94% with a kappa > 0.99) than Sentinel-2, particularly in distinguishing fragmented or intermixed habitats that Sentinel-2 could not resolve as effectively. Similarly, Li et al.^59^, in an object-based coral mapping study using 3.7 m Planet Dove imagery in the Dominican Republic, successfully classified a complex mosaic of 10 benthic habitat types (including multiple coral, seagrass, and substrate classes) with approximately 82% overall accuracy. Hedley^60^ highlighted that a spatial resolution of approximately 10 m represents the lower limit for reliably delineating many benthic features, including individual coral structure. Sentinel-2 imagery, with 10-meter pixels, operates at this threshold, often causing fine-scale habitat details to fall below the resolution limit and leading to sub-pixel mixing, where precise delineation of benthic features is required.

In mid-December 2023 we witnessed a mass *A. cervicornis* mortality event at the Coral Gardens refugium field site. The first aim of this study was to understand if changes in live *Acropora spp*. abundance at the field site were reflective of trends in the wider Coral Gardens region. We used satellite images of a 25 km^2^ area of Coral Gardens from 2011, 2017, 2021, and 2023 to model live *Acropora spp*. coral and compare data from that wider region with field data collected annually in a small area of Coral Gardens from 2012 to June 2025. Our findings reoriented our work towards looking for coral survivors. In June 2025 we pursued a reconnaissance method for validating our remote sensing model and identifying any remaining live *Acropora* spp. coral in Coral Gardens. In combination with guidance from local partners our model proved successful in identifying some *Acropora spp*. survivors. This study aimed to reexamine and build upon the original remote sensing work of Busch et al.^43^, analyze the trajectory of *Acropora spp*. decline, and search for areas of resilience in the Coral Gardens region.

## Results

### Live coral data in the field

**Fig. 2:**
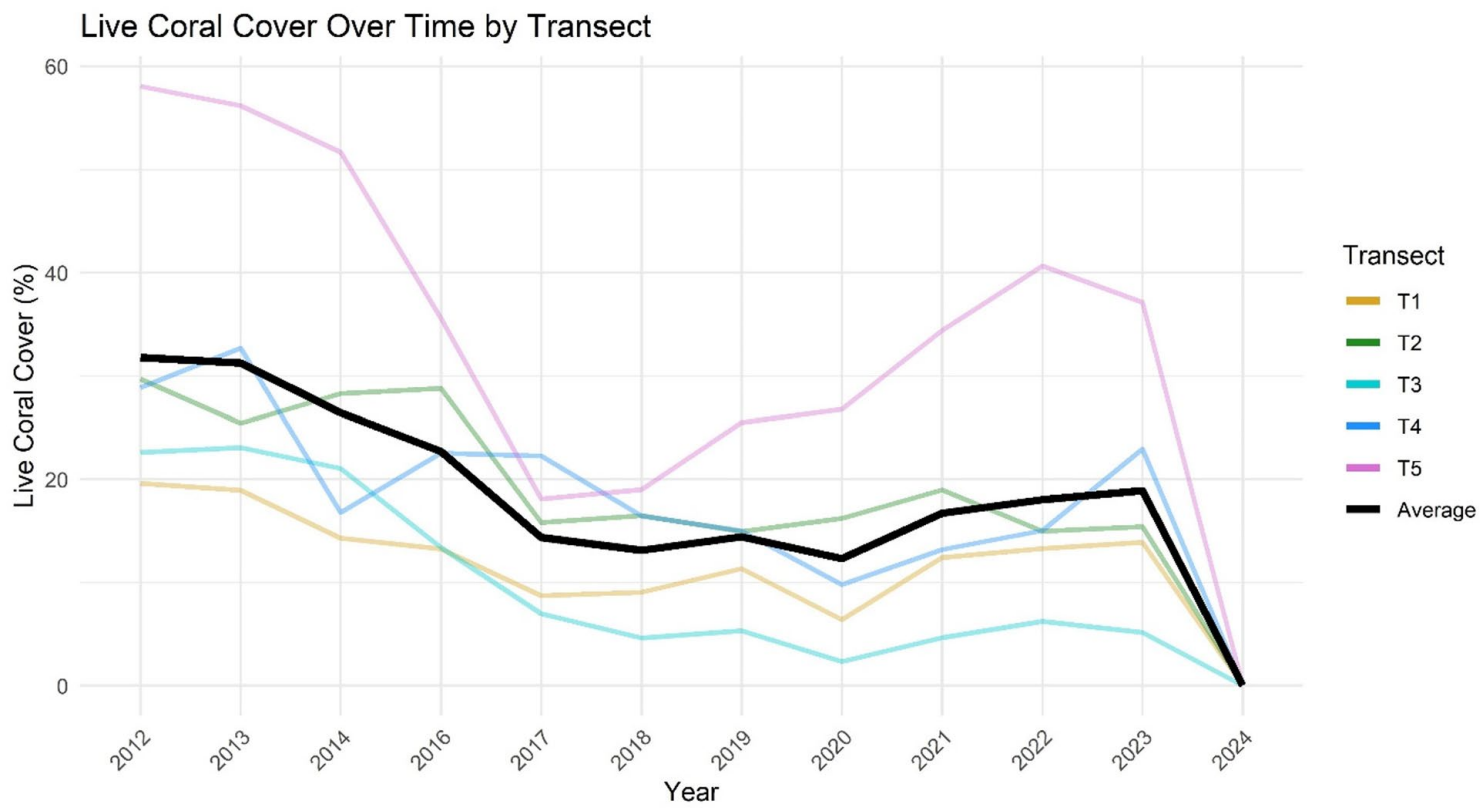
Percent live *A. cervicornis* coral abundance at the Coral Gardens field site determined by quantifying live coral tissue in ~ 146 m^2^ quadrats annually.

Live coral cover declined markedly across all five field monitoring transects between 2012 and 2024, with varying rates and patterns of change (Fig. 2). At the beginning of the time series, *A. cervicornis* cover ranged from approximately 19.6% (T1) to nearly 58% (T5). Most transects exhibited a gradual to steep decline between 2013 and 2017, with the lowest average cover occurring around 2018. Transects T1 and T3 showed the most consistent and severe reductions, with T3 dropping below 10% by 2020, although some transects showed modest recovery between 2019 and June 2023. Most notably T5 rebounded to over 40% cover, however, these gains were not sustained. A visit to the Coral Gardens field area in mid-December 2023 revealed mass mortality of *A. cervicornis* to near 0%. By 2024, *A. cervicornis* coral cover had collapsed across all transects, converging to 0%. The average trend, (smoothed black line in Fig. 2), reflects the overall pattern of initial decline, slight recovery, and a dramatic loss of coral by June 2024. The data suggest the occurrence of an acute disturbance or environmental stressor that uniformly impacted the field area.

### Coral prediction from satellite image

**Fig. 3:**
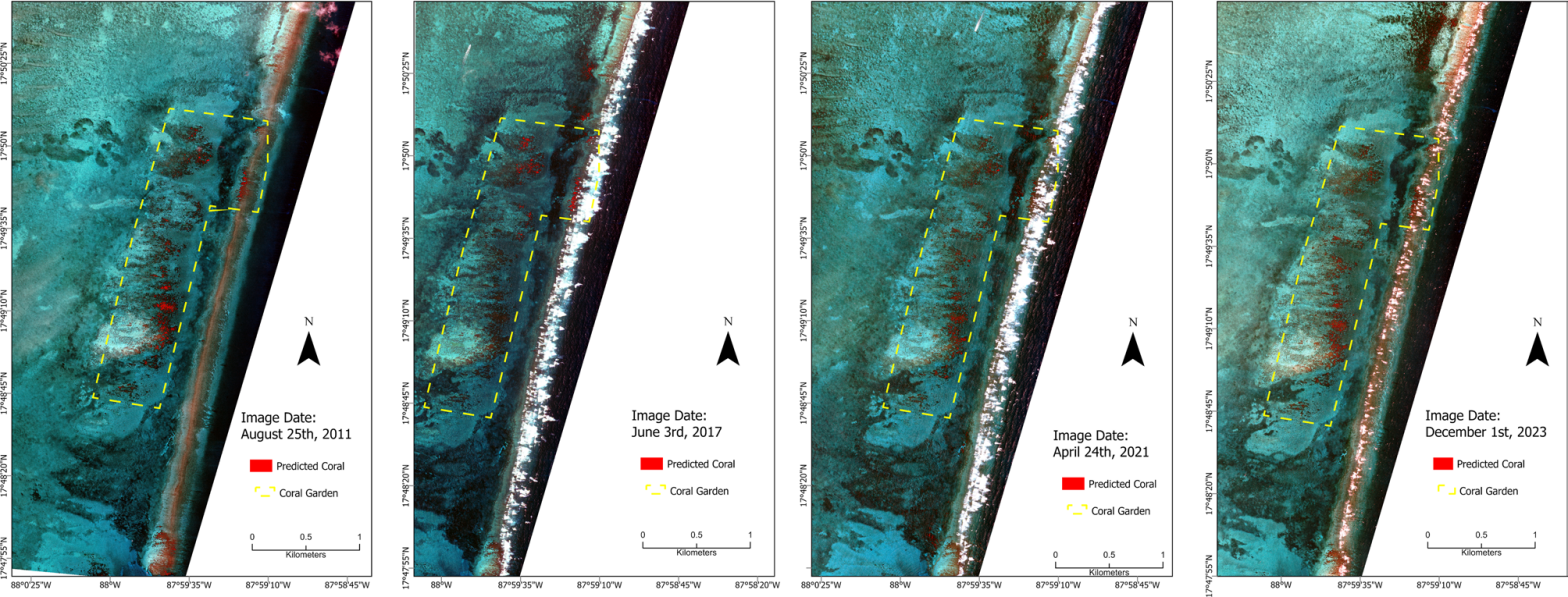
A four-panel image displaying the random tree supervised classification models created for the years 2011, 2017, 2021, and 2023. The pixels highlighted in red display predicted live coral coverage. Map generated using ArcGIS Pro version 3.3.0 (https://www.esri.com/en-us/arcgis/products/arcgis-pro/overview).

**Fig. 4:**
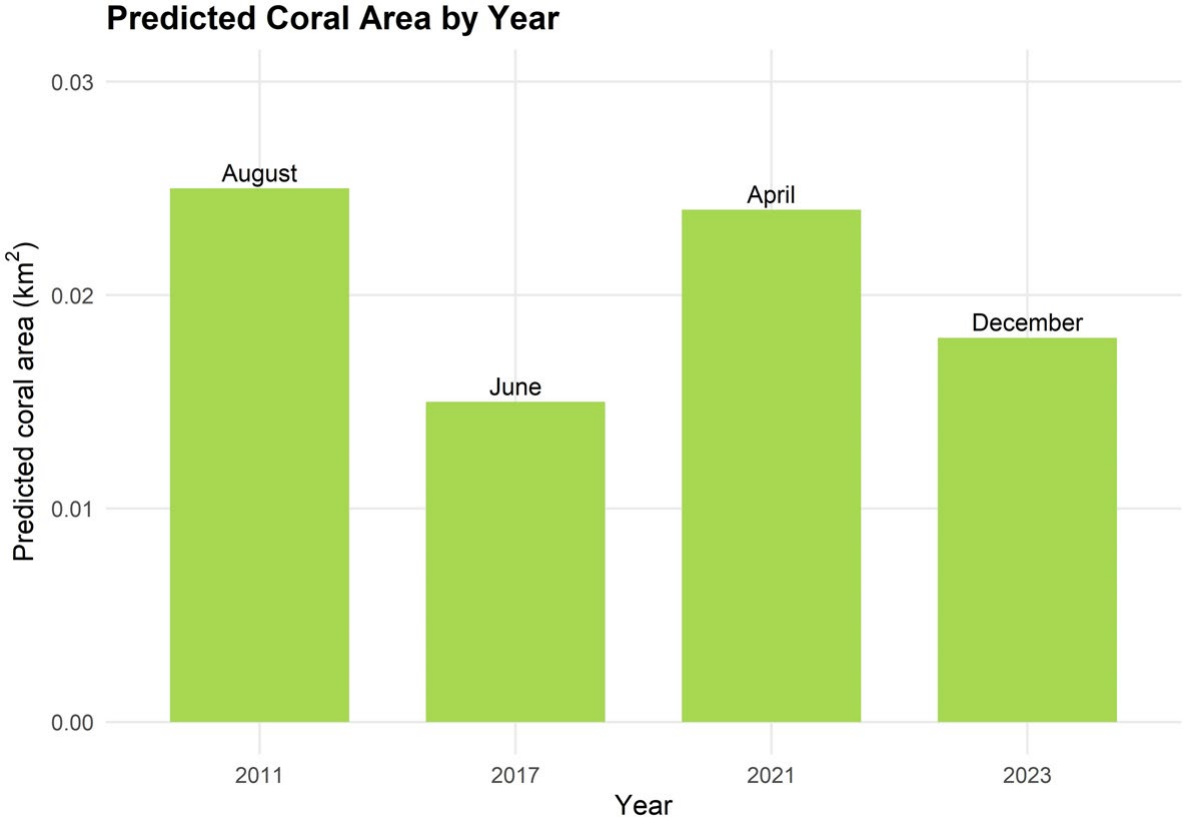
Predicted shallow-water coral area derived from satellite imagery for 2011, 2017, 2021, and 2023. Bars show the model-estimated coral area (km²) for each year, showing a decline from 2011 to 2017, a partial recovery in 2021, and a subsequent decrease in 2023.

The predicted live *Acropora spp*. coral area calculated using Random Forest Supervised Classification modeling of satellite data acquired during August 2011, June 2017, April 2021, and December 1, 2023 (Table 1), reveals substantial changes in coral populations over the 12-year period. The probabilistic threshold for classifying coral was set to 95% to minimize noise and ensure high confidence in coral predictions, thereby reducing commission errors in the final habitat maps. All four RF models for the four study years yielded overall accuracies above 95%, with sensitivity ranging from 72.92% to 95.83% and specificity exceeding 97% (Table 2). The model estimated live *Acropora spp*. coral areas of ~ 0.025 km^2^ (24,725 m²) in 2011, ~ 0.021 km^2^ (20,885 m²) in 2017, ~ 0.025 km^2^ (25,517 m²) in 2021, and ~ 0.03 km^2^ (30,052 m²) in December 2023 (Fig. 3). The 2023 model indicated an apparent increase in coral cover in the northern part of the study area. However, field verification revealed that the apparent coral signals in a northern part of the study area were false positives. The origin of the false positives is unknown but corelates with areas of seagrass beds heavily targeted by lobster fishing, as evidenced by the presence of abundant lobster traps in December of 2023. We plan to further investigate this area for the sources of this spectral signature. As a result, the predicted coral area was artificially inflated, with this effect being especially pronounced in the December 2023 model. To address this issue, the estimated coral area was refined by masking pixels within the northern region where the seagrass beds and lobster traps were observed (Fig. 4). The same masking approach was applied across all years to ensure consistency in the workflow and to better represent the real-world distribution of coral habitat. The most pronounced decrease occurred between 2011 (0.025 km^2^) and 2017 (0.015 km^2^), a period characterized by fluctuations in SSTs and the impact of Category 1 Hurricane Earl in 2016, both of which are likely contributors to coral loss (Greer et al., 2023). Notably, there was an increase in live coral area between 2017 and 2021 (0.024 km^2^), suggesting partial recovery before plunging again in 2023 (0.018 km^2^) (Fig. 4).

**Table 1:** The satellite imagery data used in this study including the name of the satellite, spatial resolution, date the image was taken, and the cloud cover percentage.

Satellite	Spatial resolution (resampled)	Image date	Cloud cover (%)
GeoEye-1	50 cm	8/25/2011	3
WorldView-2	50 cm	6/3/2017	0
GeoEye-1	50 cm	4/24/2021	0
WorldView-3	50 cm	12/1/2023	0

**Table 2:** Model evaluation metrics for Random Forest, Artificial Neural Network, and K-Nearest Neighbors models. All metrics were calculated on the independent test dataset for each model and year.

	Year	Accuracy	Kappa	Sensitivity	Specificity	Precision	Recall
Random Forest	2011	99.00	96.59	95.83	99.83	97.87	95.83
	2017	97.84	82.20	72.92	99.83	97.22	72.92
	2021	95.74	73.72	79.41	97.26	72.97	79.41
	2023	99.00	93.39	91.18	99.73	96.87	91.18
Artificial Neural Network	2011	99.38	95.50	95.83	99.67	95.83	95.83
	2017	97.69	81.53	75.00	99.50	92.30	75.00
	2021	97.24	82.08	82.35	98.63	84.85	82.50
	2023	99.00	93.21	88.24	1	1	88.24
K-Nearest Neighbors	2011	99.50	96.59	95.83	99.83	97.87	95.83
	2017	97.07	75.58	66.68	99.50	91.42	66.68
	2021	94.99	69.48	76.47	96.71	68.24	76.47
	2023	98.75	91.63	88.24	99.73	96.77	88.24

### Field reconnaissance of model prediction sites

**Fig. 5:**
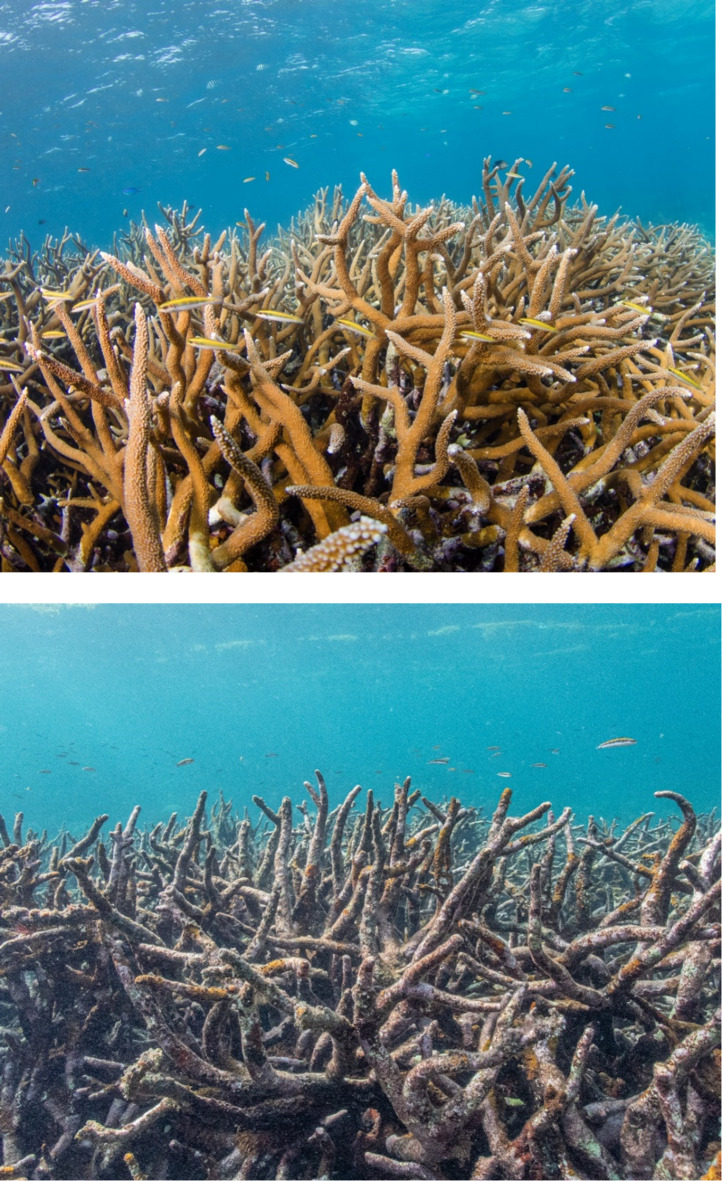
State of *Acropora cervicornis* at the T5 field site in Coral Gardens in December 2022 (top) and June 2025 (bottom). To be counted as standing, dead during field reconnaissance classification, *A. cervicornis* coral had to resemble the state of coral at the field site in June 2025, with intact framework, and similar algal cover. In the top photograph virtually all coral is alive, as indicated by the yellow, orange, and light brown hues and the bright white corallite tips. The bottom photograph shows intact skeletal framework, but no living coral tissue. Instead, the framework is coated in turf algae, red coralline algae, macroalgae, and unidentified sponges. Photos by KW.

**Fig. 6:**
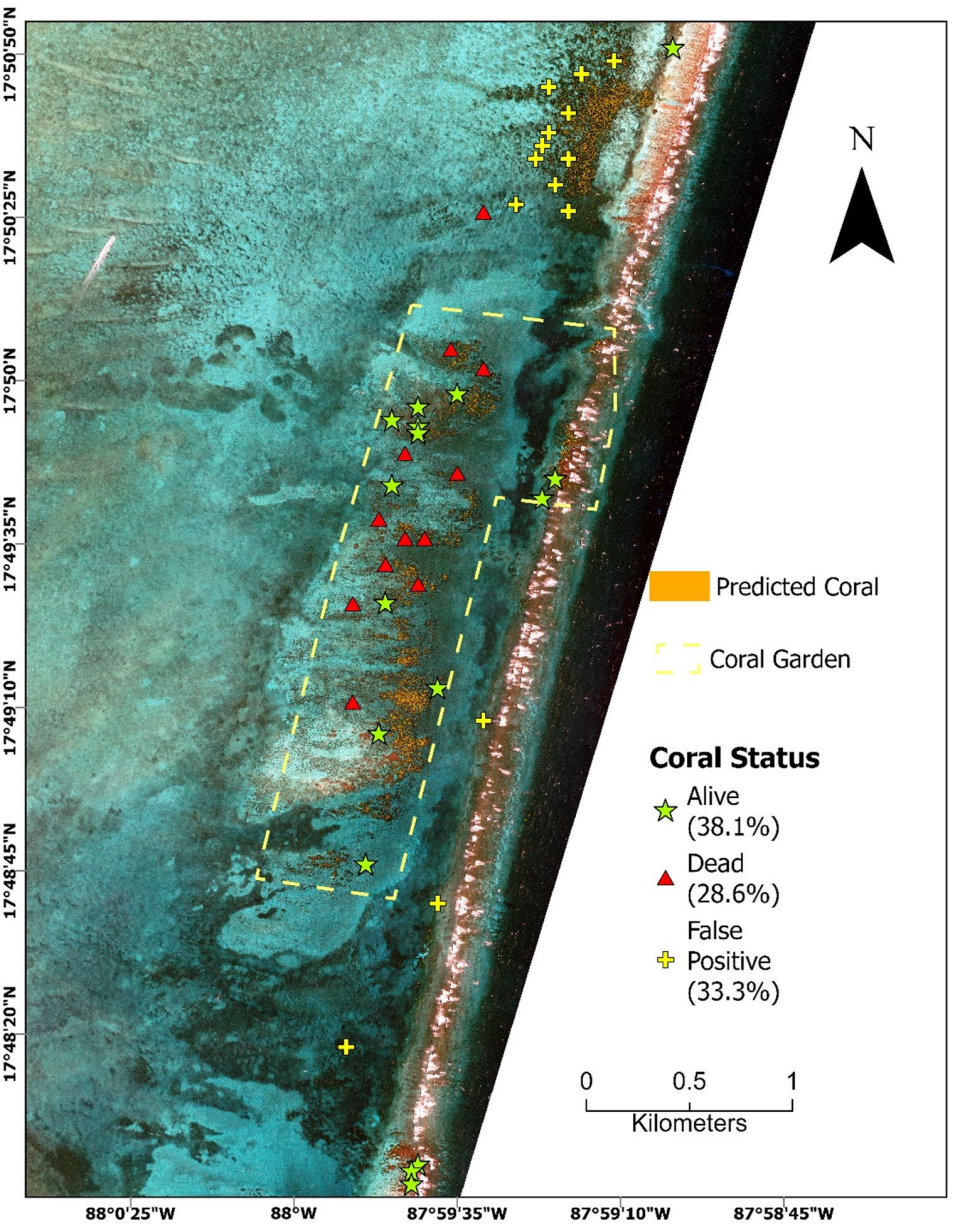
Field survey locations on the Belize coral reef used to validate model predictions (predicted coral areas shown in golden yellow). Each point represents an observed coral site, with variations in point symbols indicating live, recently dead and false positive coral locations recorded during the survey. Map generated using ArcGIS Pro version 3.3.0 (https://www.esri.com/en-us/arcgis/products/arcgis-pro/overview).

**Fig. 7:**
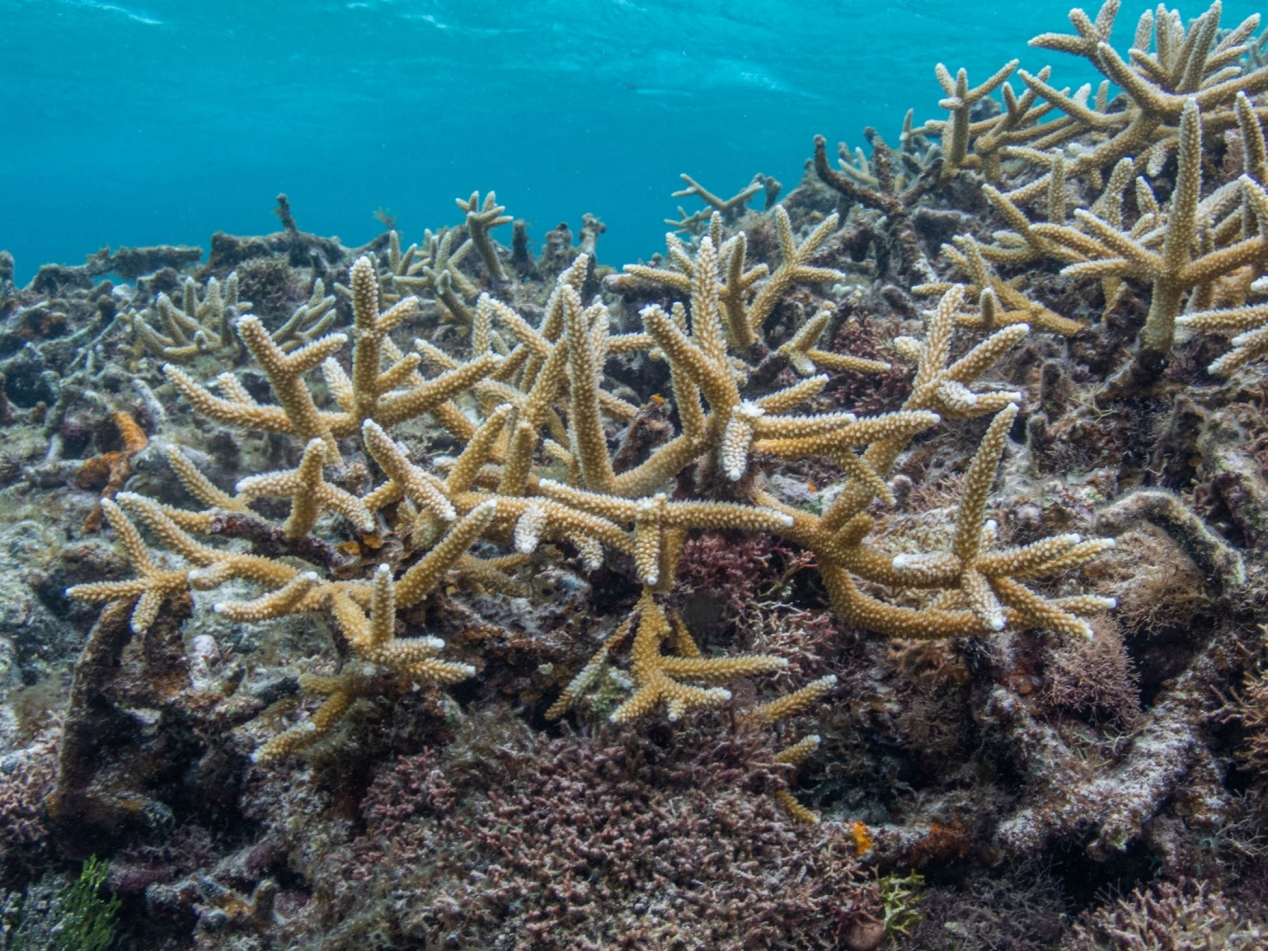
Example of live *Acropora cervicornis* found during field reconnaissance in June 2025. Note the yellow/light brown color of live coral tissue, and the bright white growing tips that are typical of this fast-growing coral, where symbiotic algae have not yet been incorporated into coral tissue. Photo by LG.

**Fig. 8:**
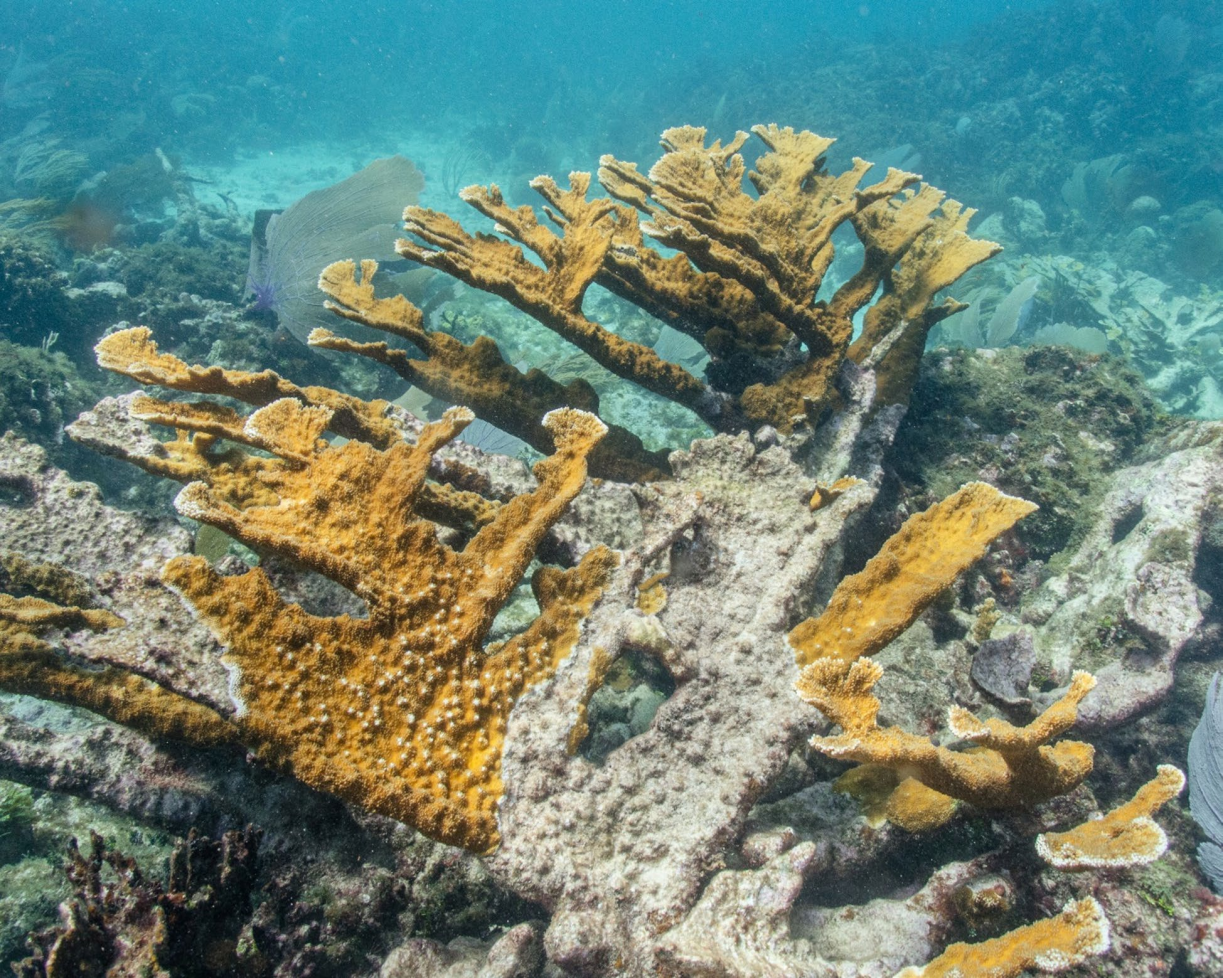
Example of live *Acropora palmata* found during field reconnaissance in June 2025. Note that this colony consists of patches of both dead (pinkish-gray and algae-covered) and live (orange-brown with bright white edges at the margins of live branches). The spotty bumps in the lower left corner of the image are ‘chimneys’ where territorial herbivorous Damselfish actively farm algae from live coral tissue which grows up as the fish nip open spots to grow turf algae. Photo by KW.

**Fig. 9:**
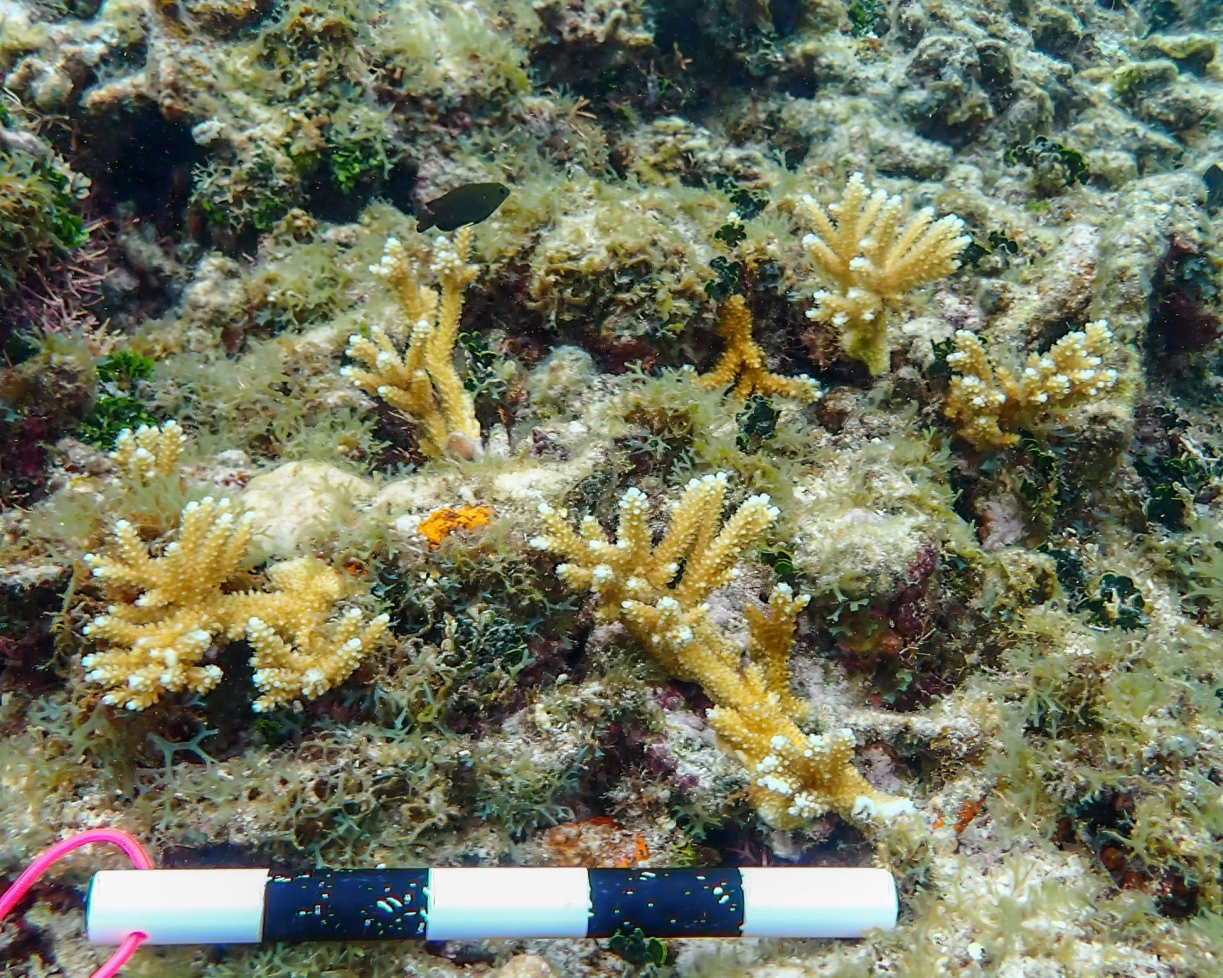
Example of live *Acropora prolifera* found during field reconnaissance in June 2025. Note this hybrid *Acropora spp*. is smaller than its sibling *A. cervicornis* and *A. palmata* parents. The scale bar measures 10 cm (2 cm per unit). While most *A. prolifera* colonies would be too small to detect with remote sensing, they are almost always found adjacent to their larger parents and at least one population in the field area known to the authors measured at least ~ 30 m across prior to the 2023 collapse. Photo by LG.

In June 2025, we conducted a field validation survey of 42 GPS locations within the modeled map area to assess model accuracy. All sites were predicted to have contained living *Acropora spp*. coral at the time of satellite data acquisition on December 1, 2023. Any live *Acropora spp*. specimens were noted as was evidence of recently dead (standing dead) *A. cervicornis* as outlined in our methods. Figure 5 shows both *A. cervicornis* coral in the field area alive prior to 2023 and the state of standing dead in June 2025. *Acropora cervicornis* that has been dead for longer time periods in this area (as observed at Coral Gardens from 2011 to 2025 by the authors) are generally recognizable as skeletal framework decays, algae bind reef material, and the delicate skeletal morphology gives way to rubble with time. Live and/or standing dead *Acropora spp.* coral were present at 28 (66.7%) of the 42 field reconnaissance sites in June 2025 (Figs. 3 and 6). Of those sites, 21 included standing dead *A. cervicornis* coral that resembled the state of coral in the Coral Gardens field area that died between June and December of 2023 (intact framework above substrate and similar fleshy and crustose coralline algae cover on coral skeletons as seen in Fig. 5). Live coral was easily identified in situ by color of live tissue and bright white growing tips (Figs. 7, 8 and 9). Only 3 sites included living *A. cervicornis* (Fig. 7). However, 16 of the 42 sites included some living *A. palmata* (Fig. 8) and 5 included small patches of *A. prolifera* (Fig. 9) coexistent with abundant turf algae. The additional 14 (33.3%) false positive sites largely contained seagrass beds and areas with multiple lobster traps in the northwestern region of the map area (Fig. 6). During ground-truthing, we visited 5 additional sites suggested by our local guide, that were previously unknown to us. One of those additional sites was predicted by the model and contained live *A. palmata*, but of the 4 other additional sites, 2 contained live *A. palmata* and were not predicted by our model.

## Discussion

This study implemented a remote sensing methodology to accurately identify live *Acropora spp*. coral with satellite imagery to document changes in live coral abundance at Coral Gardens over time after a mass mortality event. In doing so, previously undocumented *Acropora spp*. coral survivors were identified in the greater Coral Gardens region. Our Random Tree Supervised Classification model trained with spectral data from known *Acropora spp*. field sites predicted live coral populations with a degree of accuracy that allowed us to find survivors.

This study documents the massive decline of critically endangered *Acropor*a *spp*. corals over a broad 25 km^2^ area inside the Belize Barrier Reef. By mid-December 2023, Coral Gardens *Acropora spp*. coral experienced a mass mortality event, effectively decimating the population at one of the few remaining *Acropora spp*. refugia found in the Caribbean. We demonstrate that high resolution live coral data from a small field area in Coral Gardens are reflective of a much wider loss of coral from the broader region (Figs. 2 and 4). However, although live coral dropped to zero in the field area in December 2023, the Randon Forest Model revealed that some live *Acropora spp*. corals persisted in the broader region. The timing of the population decrease documented by the Random Forest model and field data show acute coral mortality following anomalous 2023 sea surface temperatures. During 2023, SSTs at Coral Gardens were irregular in several respects. SSTs exceeded 31.3 °C during October that year, the highest ever recorded by satellite for that region^61^. Heating above the bleaching threshold (MMM + 1 °C) also began earlier that year (end of May) and remained elevated longer (thru the end of October 2023). Accumulated heat stress, measured in terms of Degree Heating Weeks (DHW), exceeded 19 °C-weeks, far more than any other previous year since we began doing field surveys (1–12 °C-weeks) in that region. Our findings demonstrate that even long-serving coral refugia are no longer immune to the global coral crisis.

We, as a community, cannot preserve or protect corals that we cannot find. Locating coral survivors should remain a top priority for those hoping to safeguard endangered *Acropora spp*. corals in this once vibrant reef ecosystem, as any attempts to repopulate the reef should include careful evaluation of sites that show potential for resilient corals to persist. With the total loss of live *A. cervicornis* in our small long-term monitoring site within the Coral Gardens refugium, we found it critical to search the wider region for any surviving *Acropora spp*. corals. Our field reconnaissance, accomplished in only ~ 1.5 field days, proved that while our model did not perfectly predict the locations of *Acropora spp*., 28 of the 42 predicted locations once held extensive *Acropora spp*. coral framework and 21 of those sites appeared to have suffered extensive *A. cervicornis* mortality coincident with the 2023 marine heatwave. Most important, our model results led us to 16 previously unknown sites with at least some living *Acropora spp.* coral (Fig. 6). While most of those sites contained *A. palmata*, 5 sites contained small colonies of *A. prolifera* and 3 sites clustered behind the reef crest in the southern map area contained *A. cervicornis*.

We note that our approach facilitated discovery of all three, quite different, *Acropora spp*. morphotypes. Our model primarily identified large *Acropora palmata* stands (Fig. 8) composed of both live and recently dead framework. But the model also predicted locations composed of the smaller branching *Acropora cervicornis* (Fig. 7) and the hybrid *Acropora prolifera* (Fig. 9), even when mixed with abundant turf algae. This suggests that the model is useful for finding locations with large patches of the characteristic *Acropora spp.* spectral signature, but also areas where the live corals are less densely distributed. We suggest that these sites may be valuable as future targets for reef conservation, protection, and propagation for local coral conservation efforts^33^ that may benefit from collaborators who can locate potential coral sites from a distance.

We also note that tracking the population decline in the smaller field area at one of the largest refugia of *Acropora spp.* corals in the Caribbean had utility for describing how the broader population trends in the region reacted to continued environmental changes. If there is a known association between a small-scale reef and the broader population, monitoring can be done on the small scale and be representative of the larger population of the region. Machine learning–based classification of satellite imagery provides a cost-effective approach for tracking coral population trends across larger spatial extents and could be adapted to monitor *Acropora spp*. as well as other shallow-water coral species with distinct spectral signatures. Nevertheless, this workflow is well suited for identifying shallow-water coral habitat but may be less effective for detecting corals deeper than approximately 10 m due to limited light penetration through the water column^62,63^. Additionally, the corals being mapped must have a distinct spectral signature that is separable from surrounding benthic features. In this study, the model was effective because *Acropora spp*. corals in the region exhibit a unique spectral signature on satellite images that contrasts clearly with those of other coral species. However, it is important to recognize that area estimates obtained from classified remote sensing data inevitably inherit the residual misclassification present in any image classification, including omission and commission errors that remain beyond those quantified by our accuracy assessment. For instance, in the December 2023 predictions, the areas with abundant in-season lobster traps and seagrass beds were misclassified as coral, perhaps because of similarities in spectral response. Such false positives can significantly bias mapped coral extent by inflating area estimates and reducing classification precision. This issue can be mitigated by masking known artificial features or other areas that are prone to systematic misclassification, using ancillary data and/or targeted field observations. In this study, the northern part of the study area was masked to exclude misclassified coral pixels caused by artifacts before calculating the final coral habitat area. Importantly, these coral misclassifications were identified through field verification and incorporated into the workflow to further refine the model outputs. We therefore strongly recommend integrating field validation, at least to some extent, with satellite remote sensing to improve shallow-water coral identification. Accordingly, shallow-water coral maps derived from satellite imagery should be interpreted as best-available approximations, with greater confidence placed in relative spatial patterns and interannual differences than in absolute area estimates.

In future work, we plan to collect in situ spectral information of the dominant coral taxa using a field spectrometer and use these measurements to calibrate and refine the satellite-based models, with the goal of further reducing classification error and developing a more robust framework for predicting shallow-water coral extent. In many cases, atmospheric conditions, water clarity, and coral depth can significantly influence the spectral signature in satellite imagery, which may result in inaccuracies when predicting underwater corals from satellite data. Another key requirement is the availability of a well-documented field study area to serve as a training site for the machine learning models. Coral Gardens met this need exceptionally well, as long-term population data were available at a fine local scale over the 12-year study period. Without such known population data, accurately mapping live coral is challenging because the classification would lack defined spectral signatures for the target species. This method also depends on the availability of high-resolution satellite imagery. In this study, imagery with a spatial resolution of 50 cm or finer and minimal cloud cover was necessary to achieve reliable results.

Finally, we recognize that local knowledge of the area is fundamentally critical for maximum effectiveness in finding survivors. In the brief time we had for field reconnaissance of model predictions in June 2025, our expert local guide led us to two sites with live *Acropora spp*. coral that our model had not predicted. The wealth of knowledge available for seeking surviving corals cannot be replaced by our method. Recognizing these constraints will help refine future applications of this approach, whether by integrating complementary remote sensing techniques, expanding spectral libraries for more coral species, improving access to high-quality imagery, or better coordinating with local experts.

The current global reef crisis has been well documented in every reef ecosystem across the globe. It is important to continue to track the scale of decline, but it is increasingly important to find places where there is hope for survival. For coral conservation efforts to work, we need all hands-on deck from the lab to the remaining wild places where corals have held space. The greatest potential utility of our approach, critical to finding meaning in this work, is in providing data and fostering collaboration with local conservation efforts.

## Methods

### Live coral tissue assessment in the field

Live coral abundance was assessed annually at five permanent shallow water (< 7 m depth) transect sites in the Coral Gardens region from July 2011 to December 2023, following the protocol outlined by Greer et al.^47^. In this process, a measuring tape was deployed between two fixed rebar pins, and high-resolution photographs were taken at 1-meter intervals using a 1 m² scale frame. The best image from each quadrat was cropped and geometrically rectified in MATLAB using custom scripts developed by Stough et al. (2012). Live *Acropora cervicornis* tissue was manually segmented in Adobe Illustrator, and the proportion of live coral was quantified in MATLAB by calculating the percentage of coral pixels relative to the total pixel count in each image. Data collected from 2019 to 2023 are reported here and integrated with data previously published by Greer et al.^47^. These field-based coral abundance measurements were used to train and develop a predictive model for identifying potential live *Acropora cervicornis* habitats beyond the Coral Gardens region.

### Satellite data

High-resolution multispectral satellite imagery from the GeoEye-1, WorldView-2, and WorldView-3 satellites was used to identify live populations of *Acropora spp*. coral in the wider Coral Gardens region (Fig. 3; Table 1). Analysis-ready imagery was acquired from Satellite Imaging Corporation, having undergone orthorectification, co-registration, and preprocessing to ensure spatial accuracy and consistency across datasets. To enhance the spatial resolution of the multispectral imagery, pan-sharpening was applied, integrating the high-resolution panchromatic band with the lower-resolution multispectral bands. This process effectively improved the spatial resolution of the multispectral imagery to 50 cm while maintaining spectral integrity. A total of four satellite images from 2011, 2017, 2021, and 2023 were analyzed to assess changes in coral populations off the coast of Belize over a 16-year period. The imagery was selected to have no to minimal cloud cover over the study area (Table 1) and, where possible, to coincide with our June–July and December field campaigns; for 2011 and 2021, persistent cloud cover required the use of the closest available cloud-free scenes in time.

### Model algorithm

To identify the most suitable classifier for mapping shallow-water coral habitats, we first tested three supervised classification algorithms: Random Forest, K-Nearest Neighbors, and Artificial Neural Networks. All models were trained using the same set of training samples, and their predictive performance was evaluated on an independent test dataset using the R statistical software environment. Although all three classifiers achieved high accuracy, the Random Forest and Artificial Neural Networks models outperformed K-Nearest Neighbors and showed very similar performance across multiple evaluation metrics (Table 2). Given the well-documented success of Random Forest for mapping coral reefs and benthic habitats, we selected the Random Forest model as the final supervised classifier for delineating shallow-water corals in this study, even though the performance of Artificial Neural Networks was on par with Random Forest^64–67^. Random Forest is a non-parametric ensemble classifier that combines the predictions of many individual decision trees to produce a single, more stable outcome. Each tree is trained on a bootstrap sample of the training data, and the remaining out-of-bag samples are used to estimate internal prediction error. Final class labels for each pixel are obtained by aggregating the predictions of all trees using majority vote. By averaging over a large number of trees trained on slightly different subsets of the data, Random Forest reduces the variance and overfitting typically associated with single decision trees and provides a robust framework for distinguishing small, patchy *Acropora spp*. colonies from spectrally variable and spatially complex non-coral substrates in shallow-water environments.

### Development of the Acropora prediction model

The previously mapped live *Acropora cervicornis* population at Coral Gardens was used as the primary reference area to train the Random Forest model based on the spectral signatures of this species. In addition to Coral Gardens, a set of ground-truth locations acquired in 2011 was incorporated into the supervised classification^43^. A total of 168 points were generated within known *A. cervicornis* locations, and 2000 non-coral points were generated outside the Coral Gardens area. These samples were randomly split into 70% for model training and 30% for testing and validation. Because, in reality, coral occupies a much smaller area than non-coral substrates, a larger number of non-coral points was used to better represent this imbalance in the model. Four spectral bands from the satellite imagery, three in the visible spectrum and one in the near-infrared, were used as predictor variables for the multi-year coral prediction models.

A five-fold cross-validation was applied to the Random Forest model and repeated fifteen times to obtain stable performance estimates. Hyperparameter tuning focused on *mtry*, the number of predictor variables randomly selected for splitting at each node. The number of decision trees (*ntree*) was fixed at 1000, and the optimal *mtry* used in the final model was selected as the value that produced the highest mean cross-validated overall accuracy during model training.

### Model evaluation and field-based validation

The coral prediction models were evaluated on the independent test dataset using several standard performance metrics. We calculated the overall accuracy, which represents the proportion of correctly classified samples. We also computed Cohen’s Kappa to quantify the level of agreement between the predicted and observed classes beyond chance. Class-wise performance was assessed using sensitivity (recall) and specificity, defined as the proportion of actual coral and non-coral samples correctly identified by the model, respectively. In addition, we report producer’s accuracy and user’s accuracy for each class, where producer’s accuracy corresponds to sensitivity/recall from the perspective of the reference data, and user’s accuracy corresponds to precision, that is, the probability that a pixel classified as coral is truly coral.

To validate the accuracy of the models created, and to search for coral survivors, 42 shallow water locations (between land and the reef crest) predicted to contain live *Acropora spp*. coral were selected from the 2023 model and visited in June of 2025 over ~ 1.5 days in the field. Field validation sites were chosen for accessibility, abundance of predicted coral clusters, and limited by time in the field. Each coordinate location site was assessed by snorkelers for any evidence of live *Acropora spp*. coral or standing dead that resembled the condition of the coral that succumbed to the mass mortality event at the Coral Gardens field sites in December 2023. Field notes and photographs were taken at each location describing habitat, *Acropora spp*. present (*A. cervicornis*, *A. palmata*, *A. prolifera*), coral health, substrate, and approximate size of any living *Acropora spp*. coral. Criteria for resembling standing dead at the field sites included intact framework above the substrate and similar abundance of fleshy and crustose coralline algae coating coral skeletons (Fig. 5). In addition to the 42 model-based predicted *Acropora spp*. sites, we visited 6 additional sites suggested by our expert local guide.

## Data Availability

Data generated during the current study are available from the corresponding author on reasonable request.

## Code availability

The author(s) will make all code available upon reasonable request.
